# Optimal Reuse Design Scheduling of Mine Water Based on Improved Whale Algorithm

**DOI:** 10.3390/s22145256

**Published:** 2022-07-14

**Authors:** Yuangan Yue, Yang Liu, Lei Bo, Zihang Zhang, Hongwei Yang, Yiying Wang

**Affiliations:** 1 School of Mechanical Electronic & Information Engineering, China University of Mining and Technology (Beijing), Beijing 100083, China; zqt2000403075@student.cumtb.edu.cn (Y.Y.); liuyang@cumtb.edu.cn (Y.L.); zhangzh@student.cumtb.edu.cn (Z.Z.); zqt2100403091@student.cumtb.edu.cn (H.Y.); 2 School of Mechanical and Equipment Engineering, Hebei University of Engineering, Handan 056038, China; wangyiying@hebeu.edu.cn

**Keywords:** mine water, multi-objective optimization scheduling, improved whale algorithm, adaptive adjustment

## Abstract

The optimal scheduling of mine water is a multi-objective, multi-constraint, nonlinear, multi-stage combination of optimization problems, in view of the traditional solution methods with the increase in decision-making variable dimensions facing a large amount of computation, “dimensional disaster” and other problems, the introduction of a new intelligent simulation algorithm—the Whale Optimization Algorithm to solve the optimal scheduling problem of mine water. Aiming at the problem that the Whale Optimization Algorithm itself is prone to local optimization and slow convergence, it has been improved by improving its own parameters and introducing the inertia weight of the particle swarm and has achieved more obvious results. According to the actual situation of Nalinhe No. 2 Mine, the mathematical model of multi-target optimization of mine water is established based on the function of reuse time and reuse cost of mine water as the target function, and the balance of supply and demand of mine water, the water quality requirements of water use points at all levels, the water quantity requirements of reservoirs and the priority of water supply as the constraints. The improved Whale Optimization Algorithm was used to search optimal solution, and the results showed that the adaptability value of the improved Whale Optimization Algorithm was significantly improved compared with before, of which 8.65% and 7.69% were increased in the heating season and non-heating season, and the rate of cost reduction was 46.80% and 36.92%, and the iteration efficiency was also significantly improved, which improved the decision-making efficiency of optimal scheduling and became more suitable for the actual scheduling needs of Nalinhe No. 2 mine.

## 1. Introduction

With the rapid development of the economy, China has become the world’s largest producer and consumer of coal [1,2], although the proportion of total energy consumption of coal in China continues to decline, but it will remain the main energy source for China for a long time to come. The development and utilization of mine water are the major problems facing coal development for a long time, according to statistics, tons of coal mining produce about two tons of mine water in China [3]. China currently produces 8 billion tons of mine water per year, and the annual loss of mine water reaches 6 billion tons, which is equivalent to 60% of annual industrial and civil water shortage of China [4], but the utilization rate of mine water is only about 26% [5,6].

With the continuous development of coal mining technology and the deepening of the understanding of mine water utilization, more and more scholars began to study the treatment and utilization of mine water, and relatively large progress has been made in treatment technology and utilization model, in terms of treatment technology, Miao et al. [7] have carried out a large amount of studies on the treatment and utilization of high mineralization mine water and acid mine water, providing a technical guarantee for the use of mine water; Xu et al. [8] proposed a mine water supermagnetic separation process, with more stable treated water quality, reasonable cost and small footprint; in terms of utilization mode, Xiang [9] proposed a utilization model of the life cycle of mine water, based on analyzing and studying the characteristics of the ecological environment in central and western China proposed two kinds of mine water utilization technology models, including mine water irrigation system and mine water biological factory. Wu et al. [10] have carried out a large number of studies in the prevention and control of mine water damage and the utilization of mine water resources, and proposed the “discharge, supply and environmental protection” trinity and the five-in-one optimization of control, treatment, utilization, recharge and ecological environmental protection successively rate. However, the current utilization rate of mine water is still relatively low, mainly because the cost of mine water utilization is relatively high, the reuse rate is low, and the hierarchical classification utilization of mine water and the optimization and scheduling on this basis can solve the above problems to a certain extent, with the continuous development of the Internet of Things and cloud technology, it also provides more and more convenience for the practical application of this program [11,12], Ref. [13] established an online monitoring platform for mine water, which can better detect real-time data, Ref. [14] introduced particle swarm algorithm into the optimal dispatch of mine water, which improved the reuse rate and reuse rate of mine water to a certain extent [15], but there were fewer studies on the cost of mine water reuse. So this paper proposes a method of optimal scheduling for mine water based on an improved whale optimization algorithm, which can reduce the cost of use, improve the reuse rate, and further promote the utilization of mine water [6].

Optimal scheduling of mine water is a multi-constraint, nonlinear, multi-stage combinatorial optimization problem [16]. Traditional algorithms such as linear and nonlinear programming, dynamic programming, and step-by-step optimization algorithms are not ideal in terms of convergence, calculation rate, parameter sensitivity, etc. when facing the problem of more reservoirs or increase in dimensionality, especially for large systems with optimal allocation of water resources, the resulting “dimensional disaster” often makes the optimization method do not agree with the actual effect. With the establishment of biomimicry, the natural meta-heuristic algorithm came into being by simulating the behavioral characteristics of organisms in nature [17]. With its advantages of being easy to implement, no need for gradient information and the characteristics of avoiding trapping in local optimization and performing well, it has been widely used in the field of engineering [15,18,19,20,21,22]. The Whale Optimization Algorithm is a new heuristic algorithm based on natural inspiration proposed by Mirjalili and Lewis in 2016 [23,24]. The advantage of this global search algorithm is that it has fewer adjusting parameters, fast operation rate, and indirect reduction of the problem of parameter influence. Therefore, it has been well applied in the field of function optimization and engineering, and has good application prospects [16]. Ref. [25] applied it to the study of regional water resources optimal allocation, overcoming the problems of slow convergence speed of traditional optimization algorithms, and showing good adaptability and effectiveness in solving the problem of optimal allocation of water resources. Ref. [26] applied it to unmanned vehicle path planning, which is more feasible and effective in complex environments than quantum particle swarm algorithms and improved potential field ant colony algorithms. Ref. [27] applied it to the LED layout of indoor visible light communication, and its illuminance uniformity and average acceptance ratio were better than the genetic algorithm with faster convergence speed and better final convergence value. Ref. [28] applied it to the NP-hard combination optimization problem and achieved better results than the genetic algorithm, greedy algorithm, and random allocation algorithm. In a series of engineering applications, the Whale Optimization Algorithm has achieved good results, but it still has a slow convergence speed [29] and poor global optimization ability. In this regard, Ref. [30] introduced Logistic mapping and inertia weights to improve the Whale Optimization Algorithm and the results show that the improved whale optimization algorithm has significantly improved the calculation accuracy, which is called iterative rate, compared with the particle swarm algorithm. Ref. [31] used nonlinear and adaptive strategies for the parameters in the enveloping predatory behavior to avoid the algorithm from trapping in local optimization prematurely, and used quadratic interpolation to alleviate the problem of whale position diversity attenuation in the iterative process, so as to achieve a better position search effect. Based on the slow convergence speed of the whale optimization algorithm, Shan Wentong et al. proposed a nonlinear convergence factor to improve the traditional whale optimization algorithm, achieving a better optimal value, and it is easier to find the global optimal value [32]. Ref. [33] used an improved whale algorithm that added adaptive weight value adjustment and search strategy to extract parameters with higher precision and less iteration than the traditional whale optimization algorithm and particle swarm algorithm. Ref. [34] introduced adaptive weight, which accelerated the convergence performance of the algorithm and enhanced the global convergence of the optimization algorithm. Through the research comparison of the above papers, it is found that adjusting the convergence factor of the Whale Optimization Algorithm is conducive to accelerating the convergence speed, and the introduction of the adaptive inertia weight of the particle swarm is conducive to improving the global search ability, based on which this paper proposes to improve the Whale Optimization Algorithm with adjustment combined with the parameters of the Whale Optimization Algorithm itself and the introduction of the particle swarm inertia weighting algorithm. And the adjustment of the parameters has given full play to the advantages of the two and obtained an improved whale optimization algorithm with strong global optimization ability and fast convergence speed, which is used to find the optimal scheduling of mine water.

The main contributions of this paper are as follows: To solve the problem of low reuse rate and high reuse cost in the mine water dispatching process, a mathematical model of mine water optimization schedule is established, and the whale optimization algorithm is used to seek optimization, and at the same time, the problem of slow convergence of whale optimization algorithm and easy to fall into local optimization is improved, and it is applied to the practical application of Nalinhe No. 2 Mine, so that the cost reduction rate in the heating season and the non-heating season is 46.80% and 36.92%, respectively. The main research contents and structure of this paper are as follows: the second chapter establishes a mathematical model of optimal scheduling of mine water based on the actual situation of Nalinhe No. 2 Mine with reuse rate and reuse cost as the target function, the third chapter improves the whale optimization algorithm’s own problems, and at the same time conducts function testing and analysis, the fourth chapter applies the improved whale optimization algorithm to the actual optimization process of mine water of Nalinhe No. 2 Mine, with analysis combined with the application results, and the fifth chapter is about the research summary and prospect.

## 2. Overview and Mathematical Modeling of Nalinhe No. 2 Mine

### 2.1. Overview of the Nalinhe No. 2 Mine

The main research in this paper is based on the Nalinhe River No. 2 Mine, and the basic overview of the Nalinhe River No. 2 Mine is introduced. Nalinhe No. 2 Mine is located in Wushen Banner, Ordos City, Inner Mongolia Autonomous Region, the southernmost part of the Nalinhe Mining Area of Ordos. The mine is designed with recoverable reserves of 686.17 million tons planned production capacity of 8 million tons per year, service period of 61.3 years, and normal inrush water of 28,800 m^3^/d, maximum inrush water of 36,000 m^3^/d. If considering the yellow mud grout infiltration of 2024 m^3^/d, the normal inrush water of the mine is 30,824 m^3^/d, and the maximum inrush water is 38,024 m^3^/d. This paper follows the default normal amount of water inflow.

### 2.2. The Improvement of Traditional Invocation Pattern

**Figure 1:**
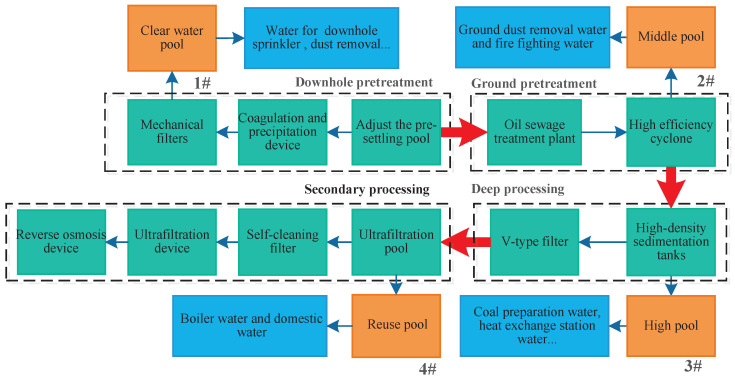
Mine water improvement dispatch flowchart.

The traditional reuse system of mine water treatment is relatively simple- being processed by all the treatment systems and then reused. The water quality testing indicates that the mine water can meet the water quality needs for the underground fire, grouting, hydraulic support, cooling, and underground dust removal after being treated by clear pool; it can meet the water quality needs for the ground dust removal and fire fighting after being treated by the intermediate pool; it can meet the water quality needs for the coal preparation, heat exchange station, cooling, green and other utility after being treated by high-level pool. Therefore, in the stages of pretreatment, secondary treatment and deep treatment, reuse outlets need to be set respectively to meet the water quality and quantity requirements in different water use points. The specific settings are as follows: there are four levels of water supply points, according to the grade of the rise are: clear water pool, intermediate pool, high-level pool, reuse pool, according to the mine water quality coupling relationship, the water in the clear water pool can be used for underground fire fighting, grouting, hydraulic support, cooling, and underground dust removal; the water in the intermediate pool can be used for ground dust removal and fire fighting, the water in the high-level pools can be used for coal preparation, heat exchange station, cooling, green, and other domestic use; the water in the multiple-use pool can be used for: domestic drinking and boiling, of which the water quality requirements of higher-grade water supply points can meet the water demand of lower-grade water points. Figure 1 below shows the improved mine water supply method. As shown as Figure 1, it shows the improved mine water supply method for the above description, and the numbers “1#–4#” indicate the node pools for scheduling.In addition, the blue part indicates where the water is used, and the cyan part indicates the pool in the water treatment process.

### 2.3. The Establishment of Objective Function of the Optimal Scheduling System

#### 2.3.1. Objective Functions

The improved invocation model of mine water, to a certain extent, has reduced the cost of the reuse process of mine water treatment, the cost of water supply to the corresponding water points that just meet the water quality requirements using nearby scheduling is the lowest, but in the actual use of the process, for some water points with high requirements for water quantity and speed, there is a problem that the water supply may be not available, resulting in serious losses, such as water for fire fighting, production, etc., so there is need for collaborative scheduling. To save costs as much as possible when the water supplies have ensued, this paper takes the reuse cost and reuse rate of mine water as the target function, and considers the quantitative and qualitative coupling relationship of mine water as the constraints, then establishes an optimal scheduling model of mine water. The details are as follows:

(1) $$min\;fit=w_{1}\left(\frac{min\sum_{i=1}^{R}\sum_{j=1}^{J}Q_{ij}\Delta t\left(Y_{i}+W_{i}\right)}{F_{max}}\right)+w_{2}\frac{min\sum_{i=1}^{R}\frac{V_{i}}{v_{i}}+\sum_{i=2}^{R}\frac{V_{i}}{V_{ci}}}{T_{max}}$$

In the formula, *R* represents the number of pools, *R* represents the month, $Q_{ij}$ represents the amount of mine water used per unit time, Δt represents time, *F* represents the cost of mine water (yuan), $F_{max}$ represents the maximum cost (yuan) in the process of mine water reuse treatment, $Y_{i}$ represents the cost in the reuse process of mine water supply (yuan/m^3^); $W_{i}$ represents the cost of water treatment at all levels (yuan/m^3^); *T* represents the time (s) of mine water treatment, $T_{max}$ represents the maximum time in the reuse process of mine water treatment, $V_{ci}$ represents the time required to purify the unit mine water (m^3^/s), $V_{i}$ represents the amount of water invoked for the *i*th pool (m^3^), $v_{ci}$ represents the speed at which the water in the reservoir is invoked (m^3^/s). The sum is the weight coefficient, which is determined according to the actual needs of the Nalinhe River No. 2 Mine, and this paper selects $w_{1}$ = 0.6, $w_{2}$ = 0.4.

#### 2.3.2. Constraints

At the same time, combined with the Nalinhe No. 2 Mine, the following constraints are established in the actual situation of the scheduling process, such as the balance of water supply, water use, and drainage, the requirements of water quality in water use points at all levels, the requirements of water transfer to the water volume of reservoirs, and the order of water supply.

(1) the balance of water supply, water use, and drainage. At any given time, the amount of inrush and discharge of mine water is balanced.

(2) $$S=\sum_{i=1}^{H}C_{i}+D$$

In the formula, *S* represents the total volume of water inrush in the mine area, *H* represents the number of water points, $C_{i}$ represents the water consumption of each water point, and *D* represents the discharge of mine water.

(2) Meet the requirements of water quality. In the process of mine water treatment and utilization, the grade of water quality after treatment is different in different treatment levels and the water point also has a certain demand for water quality, so it is necessary to meet the corresponding water quality requirements of the water point to be able to supply water.

(3) $$Z_{imin}\leq Z_{i}\leq Z_{imax}$$

In the formula, $Z_{i}$ represents the water quality requirements for treatment in phase *i*, $Z_{imin}$ represents the minimum water quality requirements when the water is transferred in phase *i*, and $Z_{imax}$ represents the highest water quality requirements when the water is transferred in phase *i*.

(3) Meet the water demand of the reservoir. When transferring water, the water in the reservoir is required to meet the needs of the water transfer.

(4) $$B_{imin}\leq B_{i}\leq B_{imax}$$

In the formula, $B_{i}$ represents the amount of water that meets the water transfer, $B_{imin}$ represents the minimum water quantity requirement when the water is transferred, and $B_{imax}$ represents the highest water quantity requirement when the water is transferred.

(4) The order coefficient of water supply. According to the actual water use of the mining area, there is a water priority: safe water > water for production > water for domestic use. The water supply priority is expressed by the water supply sequence coefficient, and the water supply sequence coefficient reflects a measure of the priority use of the water point of the kth class reservoir compared to other water resources. The reference formula for the sequence coefficient of water supply from the water source is as follows:

(5) $$q_{i}^{k}=\frac{1+n_{max}-n_{i}}{\sum_{i=1}^{n}(1+n_{max}-n_{i})}$$

In the formula, $n_{i}$ represents the serial number of the water point, $n_{max}$ represents the serial number of the water supply with the largest water source.

## 3. The Whale Optimization Algorithm and Its Improvements

### 3.1. Introduction of the Whale Optimization Algorithm

The Whale Optimization Algorithm (WOA) is a constructed mathematical model, simulating the predatory behavior of whales. The algorithm simulates the spiral bubble-net hunting of whales and forages through the mechanism of encirclement hunting, bubble hunting, and random hunting and the Whale Optimization Algorithm also includes three search methods: encirclement hunting, bubble hunting, and random variation. Assuming that the population of whales is *N* and the variable dimension of the optimized problem required for the solution is *D*, then the position of the individual *i* in the variable space is $x_{i}=\left(x_{i}^{1},x_{i}^{2},\cdots ,x_{i}^{D}\right)$, In the formula, i=1,2,…,N, and the position of each individual is a candidate solution to the problem, and the position of the optimal individual is the global optimal solution that can be found at present. The following are the three predation methods and their mathematical models.

#### 3.1.1. Encircle Predation

During their predation, whales observe the position of prey at first and then surround it. In the Whale Optimization Algorithm, the position of the leading whale is taken as the optimal solution in the current population, and all the remaining whales take the position of the leading whale as the position of prey. After determining the position of the prey, all the whales in the group will update their position in a certain way, thus forming a siege of the prey. The updated formula is as follows:

(6) $$\vec{X}\left(t+1\right)=\vec{X^{*}}\left(t\right)-\vec{A}\cdot \vec{D}$$

In the formula, *t* represents the current iteration, $\vec{X^{*}}\left(t\right)$ represents the position of the leading whale in the current iteration-the position of the optimal individual in the current iteration, representing the position of the individual after the current iteration update, $\vec{A}$ represents the convergence factor, and $\vec{D}$ represents the distance between the individual and the optimal individual for the current iteration. The the calculating formula of the convergence factor is as follows:

(7) $$\vec{A}=2\vec{a}\vec{r}-\vec{a}$$

In the formula, $\vec{r}$ represents a random number evenly distributed between [0,1], $\vec{a}$ gradually decreases linearly from 2 to 0 throughout the calculation as the number of iterations increases. The calculating formula of $\vec{a}$ is as follows:

(8) $$\vec{a}=2-\frac{2t}{N}$$

The formula for solving the distance between individual whales and the lead whale is as follows:

(9) $$\vec{D}=\left|\vec{C}\cdot \vec{X^{*}}\left(t\right)-\vec{X}\left(t\right)\right|$$

In the formula, $\vec{r}$ also represents a random number evenly distributed between [0,1], as defined in the formula for the convergence factor calculation.

#### 3.1.2. Spiral Bubble Predation

Bubble predation is achieved by spiraling through the formula. In this stage of the search, the distance between the individual whale and the prey is calculated firstly, and the distance calculation formula at this stage is as follows:

(10) $$\vec{D^{\prime }}=\left|\vec{X^{*}}\left(t\right)-\vec{X}\left(t\right)\right|$$

After that, the whale swims in a spiral path, updating its position and spitting out bubbles of varying sizes to prey. The formula of updated position is as follows:

(11) $$\vec{X}\left(t+1\right)=\vec{D^{\prime }}\cdot e^{bl}\cdot cos(2\pi l)+\vec{X^{*}}\left(t\right)$$

In the formula, *l* represents a random number between [−1,1] and *b* represents a spiral constant. As the predation mechanism of whales has two ways: encirclement predation and spiral bubble predation, in the algorithm model, the probability of these two predation methods is set at 50% each, and the updated formula of the updated Whale Optimization Algorithm can be summarized as follows:

(12) $$\vec{X}\left(t+1\right)=\vec{X^{*}}\left(t\right)-\vec{A}\cdot \vec{D}$$

(13) $$\vec{X}\left(t+1\right)=\vec{D^{\prime }}\cdot e^{bl}\cdot cos(2\pi l)+\vec{X^{*}}\left(t\right)$$

In the formula, *p* represents a random number between [0,1].

#### 3.1.3. Random Predation

During the predation process, whales will also randomly change their position according to the position of other whales, which is used to expand the search scope and try to find better prey. In the Whale Optimization Algorithm, individual whales determine whether the search scope needs to be expanded based on changes in the convergence factor $\vec{A}$. When $|\vec{A}|>1$, the individual whale will expand the search scope to search for food randomly, and carry out a global search, to avoid the algorithm trapping in local optimization and premature convergence. The random predation expression is as follows:

(14) $$\vec{X}\left(t+1\right)=\vec{X_{rand}}\left(t\right)-\vec{A}\cdot \vec{D}$$

In the formula, $\vec{X_{rand}}\left(t\right)$ represents the position of a random whale individual in the current population, $\vec{D}$ is to solve the distance between a whale individual and a random whale individual, the calculating formula is as follows:

(15) $$\vec{D}=\vec{C}\cdot \vec{X_{rand}}\left(t\right)-\vec{X}\left(t\right)$$

In this formula, the convergence factor $\vec{A}$ and the oscillation factor $\vec{D}$ are calculated in the same way as in the preceding paragraph. Then, the updated formula of the Whale Optimization Algorithm is completed:

(16) $$\vec{X}\left(t+1\right)=\vec{D^{\prime }}\cdot e^{bl}\cdot cos(2\pi l)+\vec{X^{*}}\left(t\right)$$

(17) $$\vec{X}\left(t+1\right)=\vec{X^{*}}\left(t\right)-\vec{A}\cdot \vec{D}$$

(18) $$\vec{X}\left(t+1\right)=\vec{X_{rand}}\left(t\right)-\vec{A}\cdot \vec{D}$$

### 3.2. The Optimal Scheme of the Whale Optimization Algorithm

Although the Whale Optimization Algorithm has the advantages of simple structure, few adjustment parameters, and fast convergence speed, it still has limitations in actual engineering applications, such as weak global search ability and local optimization ability, so this paper improves the above shortcomings of the Whale Optimization Algorithm.

#### 3.2.1. The Adjustment of Self-Parameter

In the optimization process of the whale optimization algorithm, *A* is an important parameter that determines the global search ability and local convergence speed of the algorithm, of which *A* is determined by the convergence factor *a*, which means the value change of the convergence factor determines the optimization quality of the algorithm. Through the review of the literature and experimental verification, it is known that when a decrease rate is slow in the early stage, it is conducive to global optimization, and the reduced speed in the later stage is conducive to increasing the local convergence rate, but in the actual iteration process, *a* is linearly decreasing, and it can not adapt to the actual optimization process. This paper takes a nonlinear exponential decrease of *a*. Original formula:

(19) $$\vec{a}=2-\frac{2t}{N}$$

In the formula, *t* represents the number of iterations, *N* represents the population size and decreases linearly from 2 to 0. Now it is necessary to improve the global optimization ability in the early stage, as well as the efficiency of search in the later stage. Therefore, it slowly becomes smaller in the early stage, and the rate of change increases in the later stage. The changed formula is as follows:

(20) $$\vec{a}=2-{\left(\frac{t}{N}\right)}^{2}-{\left(\frac{t}{N}\right)}^{4}$$

#### 3.2.2. Introduce the Improved Particle Swarm Algorithm

To enhance the local search ability, improve the convergence accuracy and accelerate the convergence speed, the paper introduces particle swarm inertia weight for improvement. A larger inertia weight is conducive to increasing the global search ability, and a smaller inertia weight is conducive to increasing the local search capability, so the paper takes the linear decreasing method, making the linear weight be correlated with the number of iterations. At the beginning of the iteration, when the linear weight is high, it is conducive to global optimization, while in the later stage, when the linear weight is small, it is conducive to the local improvement of convergence speed. The formula of inertia weight introduced is as follows:

(21) $$w=w_{start}-\left(w_{start}-w_{end}\right)\times \frac{t}{T}$$

In the formula, $w_{start}=0.9$, $w_{end}=0.4$, *t* represents the current number of iterations, and *T* is the maximum number of iterations. Therefore, the updated formula represents as follows (added inertia weight factor *w*):

(22) $$\vec{X}\left(t+1\right)=\vec{D^{\prime }}\cdot e^{bl}\cdot cos(2\pi l)+w\cdot \vec{X^{*}}\left(t\right),P\geq 0.5$$

(23) $$\vec{X}\left(t+1\right)=w\cdot \vec{X^{*}}\left(t\right)-\vec{A}\cdot \vec{D},P<0.5\cup A<1$$

(24) $$\vec{X}\left(t+1\right)=\vec{X_{rand}}\left(t\right)-w\cdot \vec{A}\cdot \vec{D},P<0.5\cup A\geq 1$$

**Figure 2:**
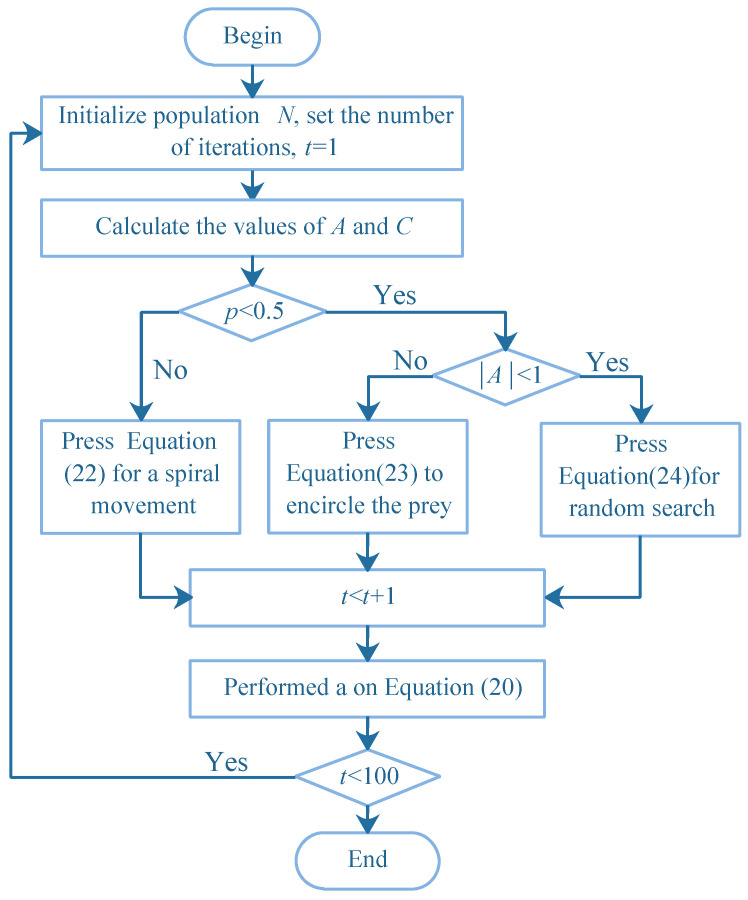
Improved flowchart of Whale Optimization Algorithm.

The improved algorithm flow is shown in Figure 2.

### 3.3. Test Functions and Analysis

To verify the convergence accuracy and convergence speed of the improved algorithm, this paper simulates the improved whale optimization algorithm and uses four classical test functions for testing. Simulate WAO (original algorithm), P-WAO (Only swarms of particles are introduced to improve the *a*), A-WAO (Only improves *a* ’s Whale Optimization Algorithm), PA-WAO (The whale algorithm that introduces particle swarm weights while improving *a*), and compare the performance of the three optimization algorithms. The population of whales is 50, the maximum number of iterations is 100, all dimensions are set to 30, and the test function is shown in Table 1 below.

**Figure 3:**
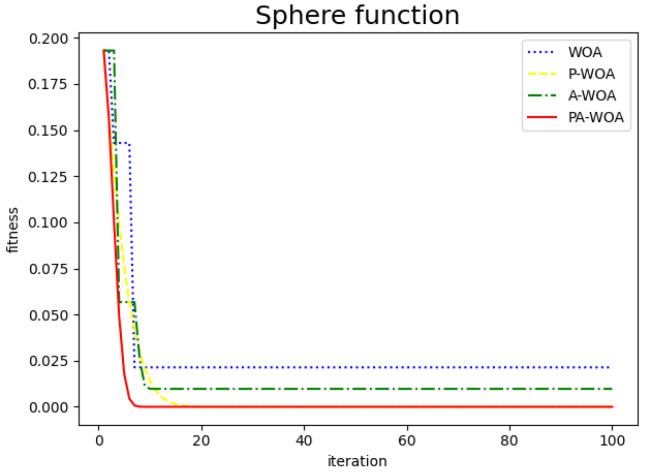
Sphere functions.

**Figure 4:**
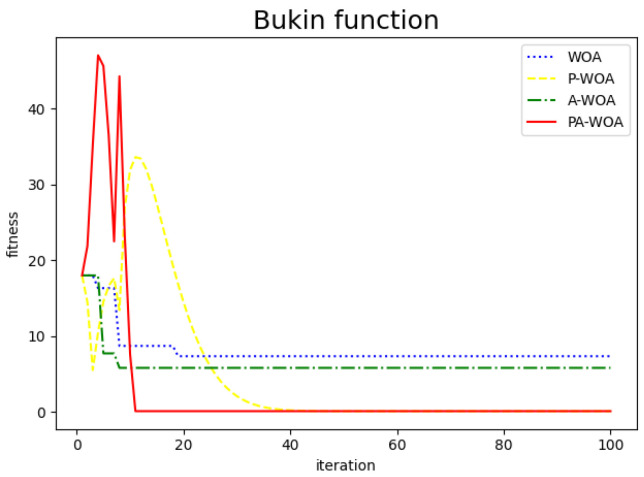
Bukin function.

**Figure 5:**
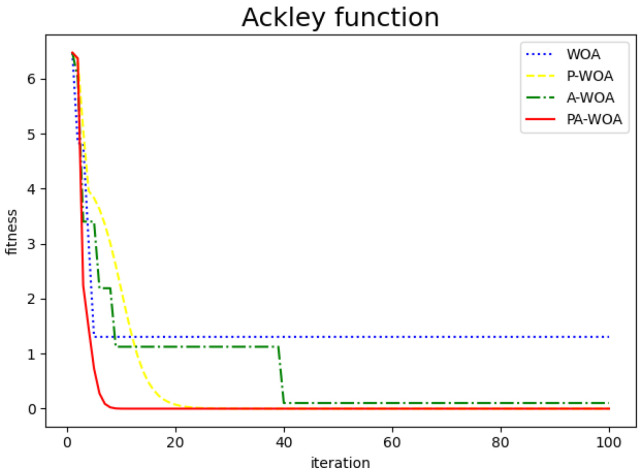
Ackley functions.

**Figure 6:**
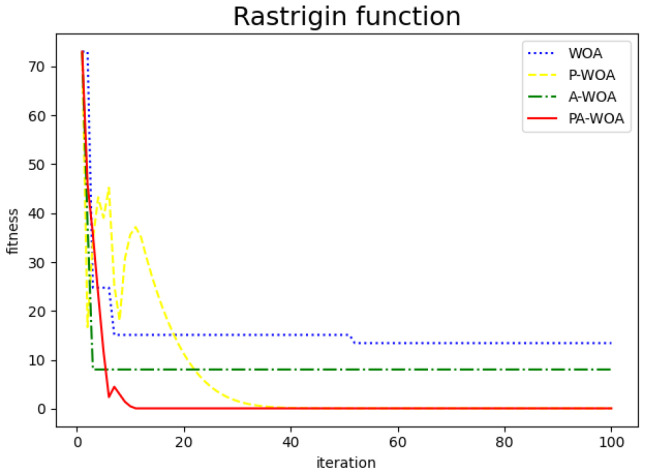
Restriqin functions.

The comparison chart of the effect before and after the improvement of the Whale Optimization Algorithm is as follows, where the blue dotted line represents the original Whale Optimization Algorithm (WAO), the yellow dotted line represents the Whale Optimization Algorithm (P-WAO) after the introduction of particle swarms, the green dotted line is the Whale Optimization Algorithm (A-WAO) that has been improved with adjustment of its own parameters of the Whale Optimization Algorithm, and the red line is the Whale Optimization Algorithm (PA-WAO) that is improved by combining the two ways. As shown in Figure 3, Figure 4, Figure 5 and Figure 6.

**Figure 7:**
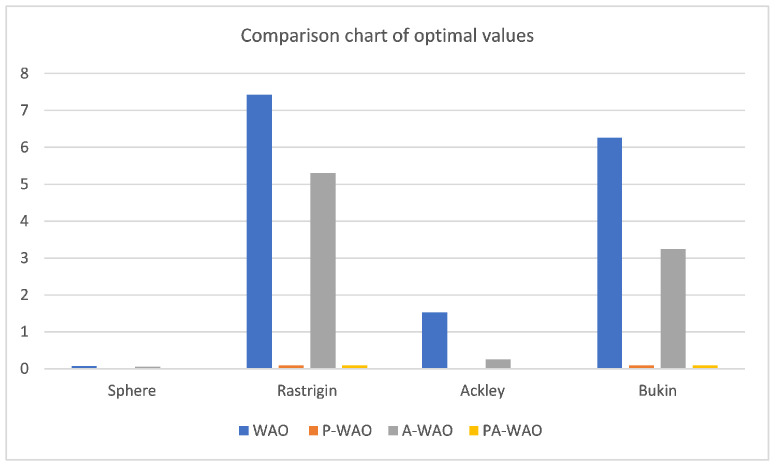
Comparison chart of optimal values.

**Figure 8:**
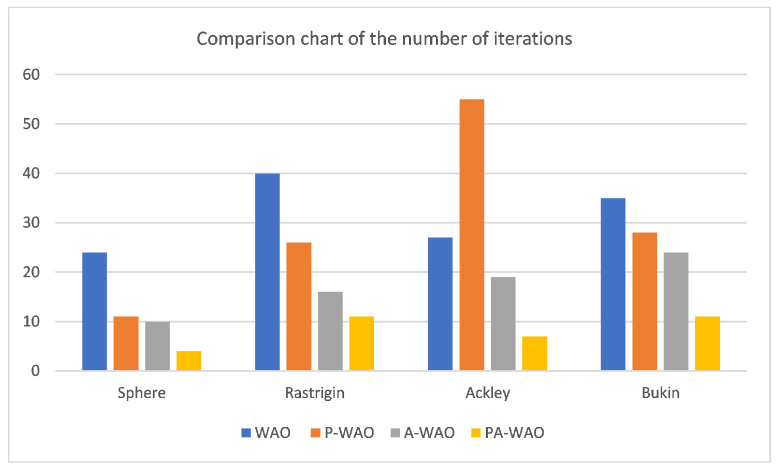
Comparison chart of the number of iterations.

To more intuitively compare the improved effects, the above four test functions are repeated with 30 random tests, then compare the average of the optimal values of the 30 iterations and the average of the number of iterations with the optimal values, which are recorded in Figure 7 and Figure 8.

As can be seen from the Figure 7: the improved P-WAO and A-WAO are better than the original WAO function in global optimization ability and local convergence speed, of which the improved P-WAO is more prominent in global optimization ability, A-WAO is more prominent in the local convergence speed, and the PA-WAO function combined with the two improved methods not only increases the global optimization ability but also accelerates the local convergence ability.

To better compare the improved effect, compare the P-WAO and PA-WAO functions that can achieve the optimal optimization value. The improved P-WAO and PA-WAO in the Bukin function, Ackley function, and Restriqin function can find the optimal value, but the local convergence speed of PA-WAO is significantly better than that of P-WAO, which can be analyzed from the number of iterations. As in the 100 test process, the number of iterations of P-WAO and PA-WAO to find the optimal value of the Reaction function was 11 and 26 times, the number of iterations to find the optimal value of the Bukin function was 11 and 28 times, the number of iterations to find the optimal value of the Ackley function was 7 and 55 times, and the number of iterations to find the optimal value of the Sphere function was 4 and 11 times, respectively. At the same time, in the test of the Sphere function, PA-WAO can find the optimal value every time, but the optimal value found by P-WAO is on average $3.2724548284812444\times 10^{-111}$, which is slightly worse than the optimization ability of PA-WAO.

In summary, PA-WAO, which improved with a combination of two algorithms, is better than P-WAO in global optimization ability and local convergence ability, which is a relatively optimal improved strategy.

## 4. Simulation Examples and Experiments

The convergence accuracy and convergence speed of the improved whale optimization algorithm have been greatly improved, and the actual water use of the Nalinhe River No. 2 Mine is now simulated. The optimization direction of the water scheduling system for the Nalinhe No. 2 Mine consists of two parts: underground scheduling and surface scheduling. Simulations were carried out by surveying the use of underground water and surface water, as well as the amount of water used in the mine area (ss shown in Table 2).

Through the optimal statistical analysis of the reuse amount of mine water at each reuse point, the comparison amount between the traditional mine water reuse model and the optimal scheduling system (nearby scheduling) model is obtained, which also includes the specific allocation of water in the heating season and the non-heating season. The results are shown in Table 3.

As can be seen from the table, after the optimization and calculation of the water consumption of the NalinHe No. 2 Mine, it can be seen through comparison that the reuse amount of the heating season under the traditional model is mainly reused by the clear water pool and the reuse pool, and the reuse amount is limited. After optimizing the scheduling system, the amount of reuse of the system increased significantly and was mainly concentrated in the intermediate pool and the high-level pool to meet the need of the water point for reuse rate, to verify the practicality of the Whale Optimization Algorithm and the improved Whale Optimization Algorithm in the optimized scheduling system of mine water, this paper will take the reuse time of mine water and adaptation value (minfit) combined with the cost as the comparison amount of original and improved algorithm, to verify the practicality of the improved algorithm.

In this paper, the population of whales is 100, the dimension is 4, and the maximum number of updated iterations is 100. The data analysis of the whale optimization algorithms and improved by the Whale Optimization Algorithm in both heating and non-warming seasons is calculated as shown in Table 4 and Table 5 combined with the Python simulation software:

As can be seen from Table 4, during the heating season, due to the multi-target reuse of the mine, the improved system has reached a reuse rate of 76.95%, which meets the water consumption of all water points in the mine. Among them, the fitness value of the Whale Optimization Algorithm (minfit) is 1.03, the fitness value of the Whale Optimization Algorithm (A-WOA), which only adjusts its own parameters, is 1.09, and the upgrade rate of fitness value is 5.77%. The fitness value of the Whale Optimization Algorithm, which introduces the particle swarm algorithm (P-WOA) and combines the two algorithms to improve (PA-WOA), and the fitness value of them is 1.12, and the upgrade rate of fitness value is 8.65%. It can be concluded that the improved PA-WOA and P-WOA have higher fitness values relative to WOA algorithms and are more suitable for the optimal scheduling of mine water. Although the cost of nearby scheduling of mine water increased by 15.4%, it was reduced by 46.80% compared with the cost of the original scheduling system, saving a total cost of 115,339.18 ( CNY) and also met the water demand of mine water. Table 5 Data analysis table of original and improved optimization system in the non-heating season.

As can be seen from Table 5, during the heating season, due to the multi-target reuse of the mine, the improved system has reached a reuse rate of 35.45%, which meets the water consumption of all water points in the mine area. Among them, the fitness value of the Whale Optimization Algorithm (minfit) is 0.78, the fitness value of the Whale Optimization Algorithm (A-WOA), which only adjusts its own parameters, is 0.82, and the upgrade rate of fitness value is 5.13%. The fitness value of the Whale Optimization Algorithm, which introduces the particle swarm algorithm (P-WOA) and combines the two algorithms to improve(PA-WOA), the fitness value is 0.84, and the upgrade rate of fitness value is 7.69%. It can be concluded that the improved PA-WOA and P-WOA have higher fitness values relative to WOA algorithms and are more suitable for the optimal scheduling of mine water. Although the cost of nearby scheduling mine water increased by 19.98%, it was reduced by 36.92% compared with the cost of the original scheduling system, saving a total of 108,889.98 (CNY) and also met the water demand of mine water.

It can be seen in Table 4 and Table 5 that the convergence accuracy of the adaptation value after the improvement of the Whale Optimization Algorithm has been significantly improved compared with before, of which 8.65% and 7.69% have been increased in the heating season and the non-heating season, which verifies the effect of the improved Whale Optimization Algorithm. At the same time, the number of iterations has also been significantly reduced, which improves the decision-making efficiency of the optimal scheduling of mine water, which is more corresponded with the actual scheduling of mine water in Nalinhe No. 2 Mine. At the same time, the improved Whale Optimization Algorithm is used to optimize the cost reduction rate of the traditional Whale Optimization Algorithm in the heating season and non-heating season, which is 46.80% and 36.92%, respectively, and the cost savings are 115,339.18 (yuan) and 108,889.98 (yuan), which greatly saves the cost and meets the needs of mine water reuse.

## 5. Conclusions

In this paper, a scheme to improve the reuse rate of mine water is proposed and verified. Firstly, the mathematical model of mine water optimization reuse schedule is established, and the optimization scheduling model of mine water is optimized by the Whale Optimization Algorithm with weak global search ability and slow convergence speed, and the improved Whale Optimization Algorithm is used to optimize the mine water optimization scheduling model. The results show that:

(1) The hierarchical and qualitative allocation strategy is adopted in the process of mine water reuse, and the intelligent optimization algorithm—Whale Optimization Algorithm is used to seek optimization, which can better solve the rate and cost problems in the process of mine water reuse and better serve the recycling of mine water resources.
(2) Given the Whale Optimization Algorithm’s own slow convergence speed and ease of falling into the local optimal characteristics, it can be solved by introducing the inertia weight of the particle swarm while adjusting its convergence factor, in which the introduction of the particle swarm inertia weight can avoid falling into the local optimal to a certain extent, improving the convergence factor can increase its local convergence rate, and pa-WAO, which integrates the two improved methods, not only avoids falling into the local optimality but also improves the convergence rate.
(3) The organic combination of mine water optimization scheduling and other solutions to mine water reuse schemes will be more conducive to solving the problems of reuse rate and reuse cost encountered in the reuse process of mine water.

## Figures and Tables

**Table 1:** Test functions.

Test Function	Function Expressions	Search Scope	Verify the Target
Sphere	$f\left(x\right)=\sum_{i=1}^{D}x_{i}^{2}$	x,y: [−100, 100]	Global search capability
Rastrigin	$f\left(x\right)=\sum_{i=1}^{D}\left(x_{i}^{2}-10cos\left(2\pi x_{i}\right)+10\right)$	x,y: [−10, 10]	Global optimization capabilities and search speed
Ackley	$f\left(x,y\right)=-20xp\left(-0.2\sqrt{0.5(x^{2}+y^{2}}\right))-\;\exp\left(0.5\left(cos2\pi x+cos2\pi y\right)\right)+20+e$	*x*, *y*: [−5, 5]	Global optimization capabilities and search speed
Bukin	$f\left(x,y\right)=100\sqrt{\left|y-0.01x^{2}\right|}+0.01\left|x+10\right|$	*x*: [−15, 5] *y*: [−3, 3]	Global optimization capabilities and search speed

**Table 2:** Water consumption in mining areas.

Mine Water Reuse Grade	Mine Water Reuse Point	Heating Season	Non-Heating Season
Down-hole treatment clear water pool	Underground fire fighting	65,735.22073	85,125.85209
	Grouting water	39,833.105	17,084.23175
	Sprinkle water down-hole to remove dust	3916.148483	8607.025781
	Cooling water	16,007.81491	20,629.249
	Hydraulic support	2321.444076	3084.392126
Pre-treatment intermediate pool	Ground dust removal	85,951.97709	77,688.64651
	Water for fire fighting	106,881.3539	77,298.12662
Secondary treatment high-level pool	Water for coal preparation	48,634.14471	36,839.152
	Water at the heat exchange station	14,866.30572	21,469.65259
	Cooling water	19,027.62374	20,063.64865
	Green water	11,815.05183	8218.430315
	Water for other utility	34,813.89783	47,655.63901
Deep treatment—reuse pool	Boiled water	80,165.00478	8252.076366
	Water for domestic use	2076.096765	1727.431289

**Table 3:** Amount of mine water reuse point reuse.

Reuse Points of Mine Water at All Levels (N)	The Traditional Model		The Optimal Scheduling System	
	Heating Season	Non-Heating Season	Heating Season	Non-Heating Season
Clear Water Pool (1#)	154,463	156,525	102,656.5	122,825.6
Intermediate Pools (2#)	0	0	169,084.9	142,435.6
High-level pools (3#)	0	0	154,848.2	152,638.6
Reusable Pools (4#)	62,735	68,526	105,455.6	15,843.8
Total reuse rate	30.75%	17.95%	76.95%	34.45%

**Table 4:** Data analysis table before and after system optimization during the heating season (Heating Season).

Model	Reuse Rate (Month)	Reuse Time (Month)	Cost (CNY)	Fitness Value	Upgrade (%)
Nearby scheduling	76.95%	638.454 h	213,556.69	-	
WOA	76.95%	537.66 h	235,945.56	1.03	0
A-WOA	76.95%	517.41 h	241,662.26	1.09	5.77
P-WOA	76.95%	501.09 h	246,451.52	1.12	8.65
PA-WOA	76.95%	501.09 h	246,451.52	1.12	8.65

**Table 5:** Data analysis table before and after system optimization in the non-heating season (non-heating season).

Model	Reuse Rate (Month)	Reuse Time (Month)	Cost (CNY)	Fitness Value	Upgrade (%)
Nearby scheduling	35.45%	487.96 h	155,060.91		
WOA	35.45%	448.08 h	176,581.82	0.78	0
A-WOA	35.45%	441.66 h	181,655.35	0.82	5.13
P-WOA	35.45%	432.41 h	186,055.67	0.84	7.69
PA-WOA	35.45%	432.41 h	186,055.67	0.84	7.69

## Data Availability

The data that support the findings of this study are available from the corresponding author upon reasonable request.
